# Parameter uncertainty in medium-term coastal morphodynamic modeling

**DOI:** 10.1038/s41598-025-02300-8

**Published:** 2025-05-27

**Authors:** Anna Kroon, Jakob C. Christiaanse, Arjen P. Luijendijk, Matthieu A. de Schipper, Roshanka Ranasinghe

**Affiliations:** 1 Department of Hydraulic Engineering, Delft University of Technology, Delft, The Netherlands; 2 Svas̆ek Hydraulics, Rotterdam, The Netherlands; 3 Resilient Ports and Coasts, Deltares, Delft, The Netherlands; 4 Water Research Laboratory, School of Civil and Environmental Engineering, UNSW Sydney, Sydney, Australia; 5 Department of Coastal and Urban Risk & Resilience, IHE Delft Institute for Water Education, Delft, The Netherlands; 6 Department of Water Engineering and Management, University of Twente, Enschede, The Netherlands

**Keywords:** Parameter uncertainty, GLUE, Process-based coastal area models, Uncertainty quantification, Coastal dynamics, (Mega) nourishment, Civil engineering, Physical oceanography

## Abstract

Rising sea levels and anthropogenic activities are intensifying pressure on coastal zones. Process-based coastal morphodynamic models are increasingly used to forecast natural and anthropogenic beach morphology changes at various spatio-temporal scales. Such predictions are crucial for the sustainable management of coasts. However, process-based morphodynamic models contain numerous free model parameters, introducing uncertainty in predictions. Systematically exploring the parameter space has remained a challenge due to the high computational demands of these morphodynamic models. Here, for the first time we quantify parameter uncertainty of a state-of-the-art morphodynamic (2DH) coastal area model (Delft3D) by systematically varying key model parameters, utilizing the Dutch national supercomputer: SurfSara. We simulate the initial (14-month) response of the Sand Engine, an innovative mega-nourishment placed along the Holland coast with 1024 strategically chosen parameter sets. The resulting simulations are analysed using *Generalised Likelihood Uncertainty Estimation* (GLUE) to attain probability distributions of morphological evolution and its sensitivity to parameter settings. The model simulations all show an alongshore redistribution of sediment resembling what is observed. However, even simulations with similar skill reveal substantial differences in predicted morphologies (same order of magnitude as the predictions’ 90% confidence interval). Our findings suggest that identifying a single optimal parameter set for coastal numerical models might be unrealistic, even for well-defined cases like large-scale coastal interventions, and that an ensemble modeling approach that quantifies parameter uncertainty is likely better suited for studies relying on morphodynamic predictions. Furthermore, we find that the magnitude of the uncertainty induced by the free model parameters is comparable to that resulting from year-to-year variations in wave climate, underscoring the importance of including both sources in uncertainty assessments.

## Introduction

The coastal zone holds important ecological, economic, and social value^1^ and is home to almost 40% of the world’s population^2^. Over the 21st century, coastal erosion is projected to threaten communities, ecosystems, and infrastructure in most regions of the world^3,4^. To understand and predict coastal change, process-based coastal morphodynamic modeling is often used. Process-based coastal models, especially those that simulate morphology in two horizontal dimensions (i.e. depth averaged coastal area models), have the potential to provide comprehensive information on coastal change at multiple spatiotemporal scales^5^. Through the inclusion of many (non-linear) processes, process-based coastal area models are thought to be able to reproduce complex coastal morphodynamics (e.g.^6–8^). The numerical outputs can be analyzed to present coastal changes through meaningful coastal indicators such as volume change and beach width. However, due to the many model parameters, their predictions may contain considerable uncertainty^9^. Understanding the impact of this parameter uncertainty can significantly improve model performance and enable better communication of expected uncertainties^10^.

Uncertainty in process-based models arises from two main sources. On the one hand, intrinsic uncertainty results from variations in environmental conditions, such as changes in wave forcing or sediment supply by rivers (e.g.^11–14^). On the other hand, epistemic uncertainty originates from uncertainties in the approach or model (parameters)^15–17^ or in data used as input or for model calibration. Process-based coastal area models simulate natural processes using mathematical equations that include free model parameters which are, at times, based on empirical knowledge. In theory, including more physics should result in a better representation of the system and thus, more accurate predictions and increased model transportability. However, increasing model complexity also increases the number of free model parameters, as each new process can easily introduce a number of additional parameters. Consequently, parameter uncertainty is an important component of epistemic uncertainty in these advanced coastal area models.

Free model parameters in process-based coastal models can represent either calibration coefficients or physical quantities. Calibration coefficients (e.g. sediment transport scaling factors) are used to tune the model for specific applications and represent subgrid-scale phenomena. The values for these parameters often have a limited physical meaning^18^. Parameters representing physical quantities (e.g. grain size, bed friction) are often site specific, space- and time-varying and therefore challenging to measure, leading to uncertain estimations^19,20^. In engineering practice, optimal values for a given model application for both types of parameters are generally obtained through a laborious manual calibration process (e.g.^21,22^). During this process, parameter values are often adjusted one at a time, based on the outcomes of previous calibration simulations. Herein, the user iteratively optimizes the model output to obtain a best agreement with observations or expectations for specific coastal indicators.

Simulation times for process-based coastal area models can easily extend to days or even weeks^5,9^, especially for simulations on medium-term (e.g. months to years) or decadal time scales. A manual calibration process exploring a significant portion of the parameter space is therefore nearly impossible, particularly when the parameters are interdependent or reciprocal (e.g.^18^). This dependency can lead to local optima for parameter settings. Consequently, the estimation of parameter values relies heavily on expert judgment and/or default values provided with the model. The subjective and time-consuming process provides little information on whether *the* optimal parameter set has been identified. Especially for models with many parameters, it is highly unlikely that one optimal parameter set even exists^18,23^.

The issue of parameter uncertainty in process-based coastal models with (many) free parameters has been illustrated for several coastal one-dimensional applications^10,18,24,25^. For instance, the influence of parameter uncertainty on prediction of the cross-shore profile was shown to affect the prediction of the crest of subtidal sandbars substantially^18^.

For two-dimensional process-based models the emphasis has often been on determining optimal parameter settings^25,26^, efficient propagation of (parameter) uncertainty^19,27^, or sensitivity analysis using small ensembles^28^. For instance, efficient local optimization tools have solely been applied for generally less computationally demanding process-based morphohodynamic models that describe the cross-shore dimension^25^. These optimization methods converge to a local optimum efficiently, and thereby only make an inventory of parameter sensitivity around the local optimum and not for the entire parameter space, making it infeasible to quantify the impact of parameter uncertainty. A more recent study used a Monte Carlo approach in combination with a coastal area model to derive parameter values that optimally reproduce observed bed levels^26^, but did not quantify the underlying uncertainty.

Here, for the first time, we explore the multi-dimensional parameter space of a process-based coastal area model to quantify uncertainty in predictions of coastal dynamics in two horizontal dimensions. Using Delft3D, we simulate the initial (i.e. first 14 months) morphological response of the Sand Engine^29^, a novel 20 million $\hbox {m}^3$ mega-sand nourishment placed along the Dutch coast (near The Hague) in 2011. The Sand Engine is well documented and has been the subject of multiple modeling studies (e.g.^5,30–34^). 1024 simulations with unique parameter sets were ran on the Dutch national computing cluster (SurfSara). The 1024 parameter sets comprise different combinations and permutations of the five most influential parameters: $f_{sus}$, the suspended sediment transport scaling, $\alpha _{rol}$, the wave energy dissipation coefficient of the roller model, $\gamma$ the wave breaking index, $\theta _{sd}$ the dry cell erosion factor, and $d_{50}$ the median sand grain size. These parameters were identified from the more than 50 parameters in the Delft3D model using a combination of expert elicitation and the Elementary Effects method. The Generalized Likelihood Uncertainty Estimation (GLUE) method was then used to quantify the parameter uncertainty in the simulations with an observation-based likelihood estimate of the model simulations. Where the likelihood of each simulation is based on the model skill, a quantitative measure of differences between the model outcome and observations.

## Results

### Uncertainty in predicted morphological changes

**Fig. 1:**
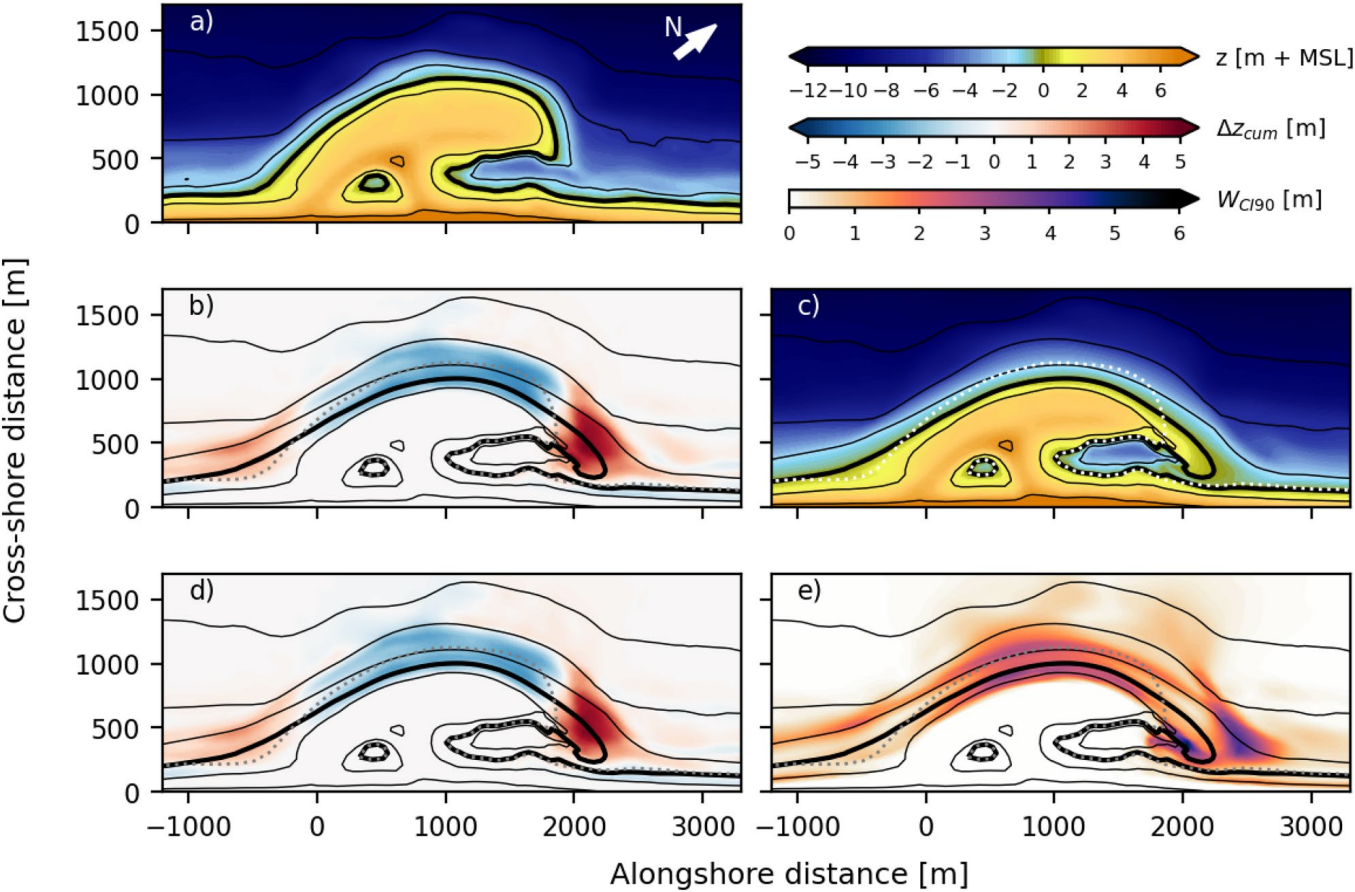
Simulation results for the acceptable Sand Engine simulations for the first 14 months. (**a**) Initial bathymetry in August 2011, (**b**) bed level changes for the simulation with the highest likelihood, (**c**) predicted bed level in September 2012 from the simulation with the highest likelihood, (**d**) median of predicted bed level changes across all acceptable runs (blue colours indicate erosion and red colours indicate sedimentation), and (**e**) width of the 90 % confidence interval ($W_{CI90}$) of the bed level changes (darker colours indicate higher uncertainty). The black contour lines are from the predicted bed level with the highest likelihood (**b**–**e**), or the initial bathymetry (**a**). The predicted 0 m + NAP depth contour is indicated with the thick black line, and the initial 0 m + NAP depth contour is indicated with the dotted line. The other contour lines depict the − 10, − 6, − 2, 2 and 6 m + NAP isobaths.

The 1024 Delft3D simulations were categorized into acceptable or unacceptable based on a behavioral threshold for skill assessment, such that approximately half of the simulations (500–600) were considered acceptable. From the acceptable GLUE model simulations, we examine several representative predictions of bed level change with respect to the initial bathymetry (Fig. 1a). Namely, the highest likelihood bed level (Fig. 1b, c), the median bed level (Fig. 1d), and an estimate of the parameter-induced uncertainty at each location in the model domain (Fig. 1e). Both the highest likelihood prediction and the median of all 1024 simulations show significant erosion at the most seaward protruding part of the beach between the -6 and 2 m isobath (relatively to local datum NAP, approximately at mean sea level). This is flanked by regions of sediment deposition on either side (Fig. 1). This sediment redistribution pattern aligns closely with observations^35^ and previous modeling efforts (e.g.^21^).

Our results show substantial uncertainty up to 6 meters in the predicted bed level changes (expressed as the width of the 90% confidence interval, $W_{CI90\%}$), with large spatial differences (Fig. 1e). The uncertainty is largest around the − 2 m + NAP isobath, the waterline of the eroding central section of the SE, and at the edges of the spit-like deposition (Fig. 1, alongshore distance $\approx$ 2000 m), with an area of relatively low uncertainty in the middle of this depositional feature. The high uncertainty around the outer perimeter of the central part of the SE indicates that the predicted erosion varies significantly in the simulations. However, the area with low uncertainty along the edge of the spit shows that all simulations indicate the formation of this sand spit. The area of high uncertainty around the tip of the spit indicates that it is mainly the length and position of the spit that varies between the different simulations. Finally, beyond the isobath of − 6 m + NAP, uncertainty rapidly decreases to low levels (< 0.25 m).

The central nearshore part of the SE displays both the highest bed level change and the highest uncertainty between the acceptable simulations, and a positive correlation is found between uncertainty bandwidth ($W_{CI90}$) and bed level changes ($r^{2}$ = 0.42). However, uncertainty can also be high in places where predicted bed level changes are small. For example, just north of the spit area, the area with a large confidence interval near the tip of the spit (x $\approx$ [2400, 2700]), extends about 200–300 m further alongshore (Fig. 1e) than the zone where high accretion is expected (Fig. 1d, x $\approx$ [1900, 2400]).

**Fig. 2:**
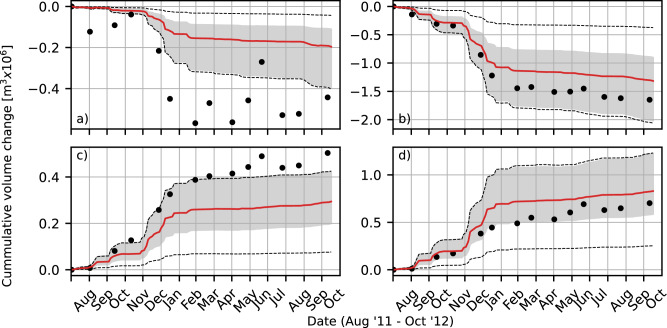
Observed (dots) and predicted volume changes in time for (**a**) the entire Sand Engine, (**b**) the Central section, (**c**) the South section, (**d**) and the North section. The grey-shaded areas indicate the 90% confidence interval of the 500+ acceptable simulations. The solid lines indicate the predicted median values. The dotted lines span the range of all 1024 simulations. Note that the range on the vertical axes vary between panels. The location of the different sections are depicted in Fig. 6b.

The uncertainty in predicted bed levels translates into uncertainty in coastal indicators such as the sediment volumes. The sediment volume in the central placement area (Fig. 2b) shows a decrease of 1.3 [0.9 2.0] (median [5%-percentile 95%-percentile]) million ${\text{m}}^{3}$ after the 14-month simulation period. Concurrent accretion at adjacent coastal sections South (Fig. 2c) and North (Fig. 2d) amounts to 0.29 [0.19 0.41] million ${\text{m}}^{3}$ and 0.83 [0.60 1.24] million ${\text{m}}^{3}$, respectively. Hence, uncertainty is largest for the central section ($W_{CI90}$ = 1.1 million i.e., ${\text{m}}^{3}$, 85% of the median), followed by the North section ($W_{CI90}$ = 0.63 million i.e., ${\text{m}}^{3}$, 76% of the median). The predicted confidence intervals are asymmetric around the median, with larger intervals for higher magnitudes of change, this suggests that the underlying parameter uncertainty is non-Gaussian.

The presented uncertainty in the predicted coastal changes is influenced by the incident wave energy. Initially, the magnitude of volume change are largest, coinciding with high-energy events (i.e., storms) in October, December, and January (Fig. 2). After the first seven months, the increase/decrease is more moderate, a model result that is in accordance with observations^35^.

### Parameter sensitivity and optimization

**Fig. 3:**
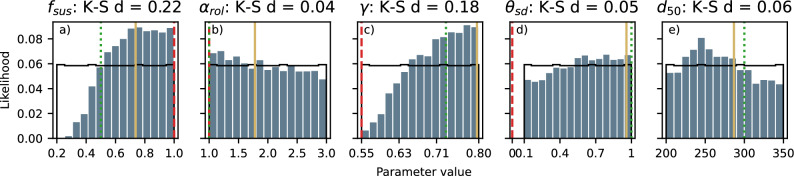
Prior (black line) and posterior distributions (histogram bars) for the five input parameters (**a**) $f_{sus}$, the suspended sediment transport scaling, (**b**) $\alpha _{rol}$, the wave energy dissipation coefficient of the roller model, (**c**) $\gamma$, the wave breaking index, (**d**) $\theta _{sd}$, the dry cell erosion factor, and (**e**) $d_{50}$, the median sand grain size examined in the GLUE analysis together with the Delft3D default values (red, dashed line), calibrated parameter values used by Luijendijk et al.^5,21^ (green, dotted line), and values for simulation with the highest combined likelihood (yellow line).

The model’s uncertainty and sensitivity to different parameters can be examined by comparing the prior (uniform) and posterior distributions (Fig. 3). For parameters $f_{sus}$ (the suspended sediment transport scaling), $\gamma$ (the wave breaking index), and $d_{50}$ (the median sand grain size), the posterior distribution shows a clear variation from the prior uniform distribution across the explored parameter space (Fig. 3a, c, e). Higher values of $f_{sus}$ and $\gamma$ have a higher likelihood, indicating that the model performs better when sediment transport scaling is high and wave breaking is initiated at smaller water depths (i.e., a more concentrated surf zone). The distribution of $d_{50}$ shows a peak at 240-250 $\mu m$. The degree of variation between both distributions (quantified using the Kolmogorov–Smirnov K–S distance, i.e., the largest distance between cumulative distributions^36^), quantifies the influence on the model’s outcome and prediction skill. High K–S distances ($\approx$0.2) are found for $f_{sus}$ and $\gamma$, showing that small variations in these parameters have a large impact on model outcomes.

Using parameter values from previous studies with the Delft3D model suite, even those focused on the same coastal site, does not result in simulations with the highest predictive skill (comparing green and red with yellow line in Fig. 3). The calibrated values for $\gamma$ and $\theta _{sd}$ (i.e., the dry cell erosion factor) as presented by Luijendijk et al. (2017)^5,21^ deviate significantly from the Delft3D default parameter settings^37^, but are supported by our posterior distributions, which show a higher likelihood around calibrated values of Luijendijk et al. This is not the case for parameter $f_{sus}$, which clearly shows a higher likelihood for values larger than the calibrated settings of Luijendijk et al. Similarly, the likelihood for $d_{50}$ is highest for lower values than the $d_{50}$-value as used by Luijendijk et al.

The posterior distributions can also be used to identify optimal parameter settings, although this can be difficult due to parameter interdependence^18^. Selecting the highest likelihood value for each parameter individually will only result in the simulation with the highest skill, if all parameters are independent. This is not applicable to the Delft3D coastal area model and our simulations. In case of parameter dependence, optimal parameter sets can be approximated by selecting a value for one parameter and conditioning the posterior distributions of the other parameters to a small range around the selected value of the fixed parameter. If the sample size is sufficiently large, this process can be repeated for each parameter, creating new conditional distributions for the remaining ones.

Considering our sample size of acceptable runs ($n = 507$), we only condition the distributions once and choose the most likely setting for the remaining four parameters. To ensure a reasonable sample size (50–100 samples), we create the conditional distributions from all parameter sets for which the value of the fixed parameter deviates by a maximum of 5% from the selected optimal value. We do this three separate times for the three most influential parameters ($f_{sus}$, $\gamma$, and $d_{50}$), leading to three different sets of parameter values that be considered optimal ($OPS_{1}$, $OPS_{2}$ and $OPS_{3}$). Another set ($OPS_{4}$) is obtained by manually selecting parameter values that contain most high-likelihood simulations. The final set ($OPS_{5}$) is simply obtained by selecting the simulation with the highest combined likelihood among all 1024 simulations. All OPS simulations have a higher model skill than the prediction of Luijendijk et al.^5,21^.

**Fig. 4:**
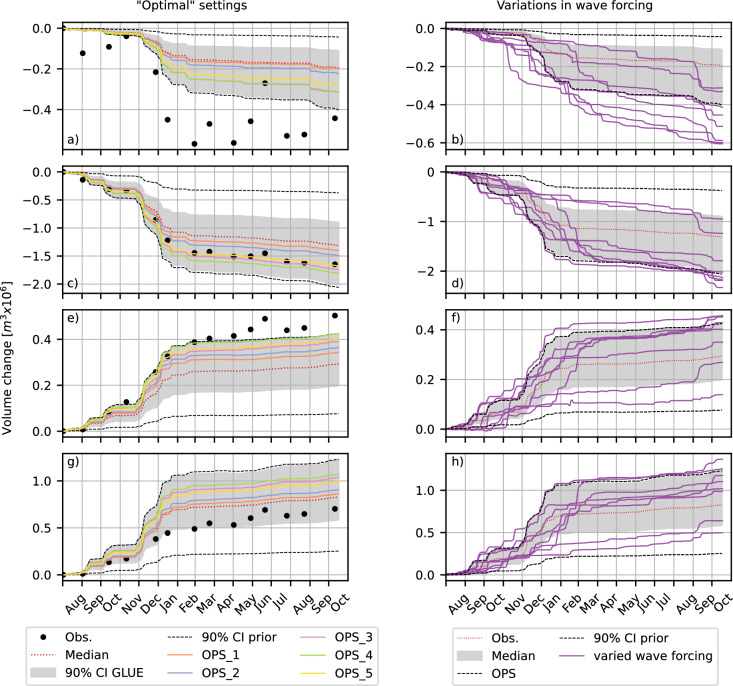
Time series of cumulative volume changes, showing the observations (black dots), 90% confidence interval (grey area) and median of the predicted volume changes (red, dotted line), apriori 90% confidence interval (black dashed lines). For the entire SE (**a**, **b**), Middle section (**c**, **d**), South section (**e**, **f**) and the North section (**g**, **h**). In the left side panels (**a**, **c**, **e**, **g**) a comparison is made with the optimized parameter values (OPS1 to OPS5) shown with the solid, pastel coloured lines. In the right side panels (**b**, **d**, **f**, **h**) a comparison is made with variations in wave forcing shown with the solid, purple lines. Note that the range on the vertical axes vary between panels. The location of the different sections are depicted in Fig. 6b.

Although the five *OPS* simulations have similar model skill, the results differ substantially. For example, the predicted cumulative volume changes span 40-50% of the 90% confidence interval (i.e., the uncertainty range, Fig 4a, c, e, g).

### Free model parameter importance

To examine the significance of the quantified model parameter uncertainty, we make a comparison to the variations in the simulations caused by intrinsic uncertainty, such as inter-annual variations in wave forcing. Intrinsic uncertainty has been considered important in morphological modeling^11–13,15^, and an important source of uncertainty in simplified one-line modeling^20^. To that end we compare the predicted bandwidth to several additional simulations where wave forcing is varied while keeping the parameter set constant. The results show that average and high wave energy years can result in a 25–30$\%$ variation in simulations (Fig. 4b, d, f, h), but calm years can reduce the predicted volume changes by more than half (Fig. 4b, d, f, h). These simulations demonstrate that both the uncertainty in free model parameters and year-to-year variability in wave climate can significantly impact the simulation results over this timescale. Therefore, both factors should be considered when predicting coastal scenarios similar to the current test case.

## Discussion

Process-based morphodynamic modeling of coastal zones has traditionally been subject to uncertainty. Predictions of sediment transport, for instance, can easily vary by an order of magnitude due to the empirical nature of current underlying formulations^38,39^. While process-based models have improved significantly over the last decades, and are capable of simulating morphodynamic changes in two horizontal dimensions for multiple years^6,7,40^, our results show considerable bandwidth comparable to the simulated median values. This level of sensitivity is likely present in a variety of coastal settings. New model approaches may be needed^9^ and may assist to reduce this uncertainty. In the meantime, more frequent incorporation of quantitative uncertainty information with model outcomes is important, especially when presenting model outcomes to decision makers. Clearly communicating uncertainties can enhance the understanding and application of model results.

Our results also reveal considerable spatial variation in the uncertainty bandwidth, which translates to the uncertainty in aggregated coastal indicators used to describe morphological development. The bed level near the waterline and in the spit area is especially subject to large uncertainty (Fig. 1), which will translate into uncertainty in coastal indicators focused on these areas (e.g. shoreline position). This sensitivity highlights the importance of carefully selecting coastal indicators and the spatial subdomains for calibration. Parameter values that yielded the best skill for one metric are not best for another, illustrating that a universally optimal set of parameter values is unlikely to exist. Depending on the coastal state indicator or metric of interest, the impact of the input parameter may also vary. If interested in a local variable (e.g. shoreline position) or limited area, sensitivities may not be apparent when calibrating on a global variable (e.g. bed level). This underlines the importance of using similar indicators for calibration and desired predictions of morphodynamic response.

Parameter uncertainty in our study is significant compared to the impact of variations in wave forcing. This relative importance of parameter uncertainty and model errors is expected to increase over time^20,41^. Moreover, parameter uncertainty and wave forcing variations may be correlated^24,42^. Although our results do not display how parameter settings and forcing are correlated, they show that the growth of uncertainty in predicted values is linked to the magnitude of bed level changes, and incoming wave energy. This suggests that this correlation is likely present in our findings as well.

The impact of uncertainty in model parameters is especially important when using the model to forecast into the future. For data-rich environments the GLUE method can be used to optimize parameter values. With this systematic approach we achieved an increase in model skill compared to the skill obtained with a one-at-a-time calibration^5^. However, considering the limited number of samples and the correlations between several parameters, it remains challenging to identify optimal parameter values. Even in our case study several near-optimal parameter sets could be identified with high skill. As there are numerous different parameter sets resulting in a similar model skill, this confirms the model’s equifinality (multiple settings can lead to similar results). Without the constraint of observation data, models with comparable skill for a calibration period can diverge over the longer term for other prediction periods^43^. In some cases, the uncertainty in predictions will be physically bounded^14^ such as by sediment availability or maximum bed slope. Nonetheless, given the large uncertainty in model outcomes, it would be prudent to include an ensemble of parameter sets or model approaches in studies relying on morphodynamic predictions. Although it may be difficult to replicate our approach at other locations, the insights gained about parameter sensitivity and prediction uncertainty can help streamline modeling strategies in practice. Even when computational resources are limited the key principles of ensemble modeling, such as selective parameter testing and not not relying on single deterministic simulations, can be effectively applied to enhance robustness and reliability of any modeling study, anywhere in the world.

## Conclusions

We explored the multi-dimensional parameter space of a coastal area model to quantify uncertainty in predicted coastal dynamics that follow from the uncertainty in the model parameter values. An extensive set of parameters is considered and systematically reduced to five key parameters ($f_{sus}$, the suspended sediment transport scaling, $\alpha _{rol}$, the wave energy dissipation coefficient of the roller model, $\gamma$ the wave breaking index, $\theta _{sd}$ the dry cell erosion factor, and $d_{50}$ the median sand grain size). Uncertainty resulting from these five parameters was quantified using 1024 morphodynamic Delft3D computations of the initial (first 14-month) evolution of the Sand Engine mega-nourishment located along the Dutch coast.

Our results show, the parameter uncertainty translates into significant uncertainty in simulated coastal changes. The simulations have the widest confidence intervals at locations and times when the bed level changes are largest. For the Sand Engine, this translates into wide confidence intervals in the dynamic spit area and around the -2 m + MSL isobath. The resulting uncertainty distributions are non-Gaussian are skewed towards larger changes. As such, multivariate normal distribution functions commonly used to estimate parameter uncertainty cannot adequately account for the correlated and non-Gaussian nature of the input parameters in these models.

In our application, the suspended sediment transport scaling ($f_{sus}$), the breaker index ($\gamma$), and the grain size ($d_{50}$) contribute the most to the overall uncertainty in the Delft3D morphodynamic predictions. Because the investigated parameters are partially correlated, finding true optimal parameter settings or confirming the existence of a single optimal set is challenging. Several near-optimal sets are identified by constraining the other parameters to the most optimal value for the three most influential parameters. Still, none of these optimal predictions result in a skill higher than the highest skill among all 1024 simulations. All three sets outperform the manually calibrated reference computation^21^. Across the different parameter sets with very high and comparable skill, simulated coastal changes can be substantially different (40–50% of the 90% confidence interval). This highlights the equifinality in the model and the need for validation rather than further optimization to prevent overfitting.

These findings suggest that identifying a single optimal parameter set for medium-term (months to years) 2DH coastal morphodynamic model applications is unrealistic, even for a case with a strong morphological signal such as the Sand Engine. Instead, it might be prudent to employ an ensemble approach, especially in cases where the morphological signal is weak. Furthermore, we find that the magnitude of the uncertainty induced by the free model parameters is comparable to that associated with year-to-year variations in wave climate at the Sand Engine. These results underscore the importance of including both sources (parameter uncertainty and variations in wave forcing) in uncertainty assessments, although the relative contribution from each will likely depend on the local geomorphological and environmental settings.

## Methods

Our simulations are replicating the morphological development after the implementation of a large intervention in the coastal zone, the Sand Engine mega nourishment^29^ implemented in 2011 along the Dutch Delfland coast. The large perturbation presents an ideal test case to reveal how parameter uncertainty influences coastal area predictions, as it induces large variations in bed level that exceed both natural variability and measurement errors in observed response. The Sand Engine is a well-monitored nourishment for which high-resolution spatio-temporal observation data is available^44^. The data shows a redistribution of sediments in the alongshore direction^35,45^. We carried out 14-month morphological simulations using the Delft3D model suite and the schematization of^5,21^ to reproduce the observed redistribution of sediments between August 2011 and October 2012. This model period covers the initial strong adaptation of the nourishment, which was measured with a high temporal resolution (monthly), but longer simulations can be found in Luijendijk et al.^5^. We obtained 1024 unique simulations with varying values of the five most influential free model parameters, identified using the Elementary Effects method, to make an observation-based estimate of the parameter-induced uncertainty in the simulations, using the Generalized Likelihood Uncertainty Estimation (GLUE) method^46^. The five parameters were selected based on literature review, expert solicitation, and sensitivity analysis^47^.

### Sand engine nourishment

**Fig. 5:**
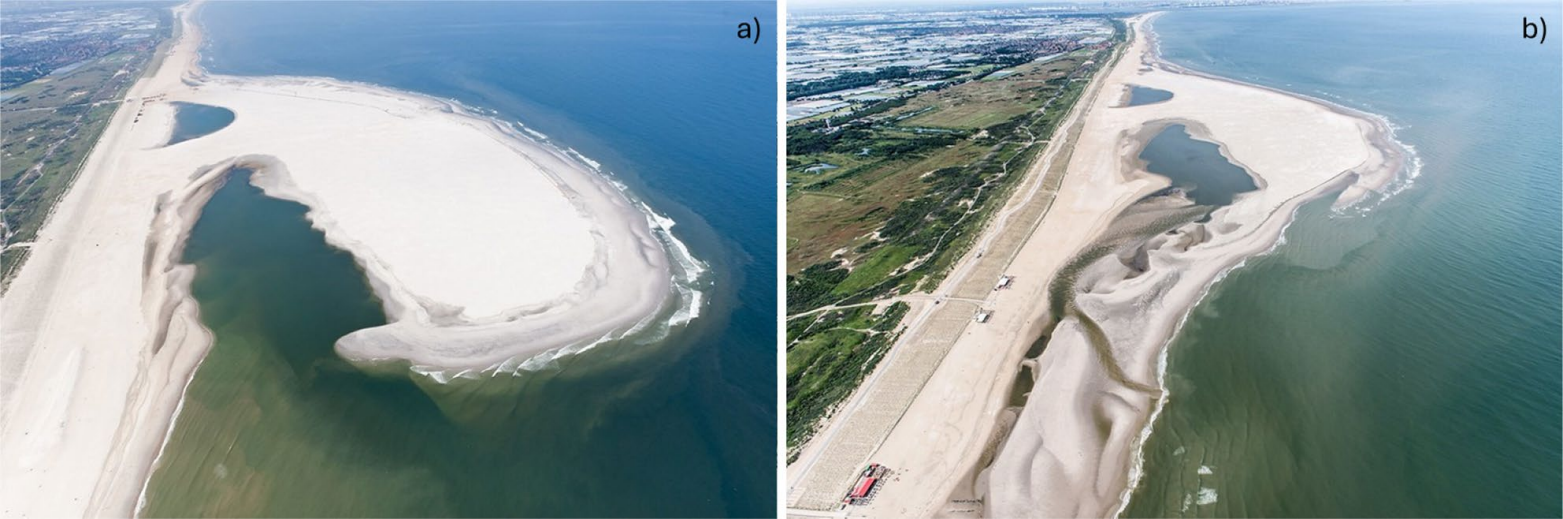
Aerial photos of the Sand Engine peninsula, taken from the North in (**a**) July 2011 (shortly after construction) and (**b**) July 2012 (after the first year). *Source*: Rijkswaterstaat and van Houdt (2012)^76^.

The Sand Engine (SE) was implemented at the sandy Delfland coastal cell in the Netherlands in 2011 (Fig. 5). The nourishment was shaped as a large hook-shaped sand peninsula, flanked by two shoreface nourishments, and measured approximately 2.4 km in alongshore and 1 km in cross-shore direction after construction. The SE was expected to feed the adjacent coastline over 20–30 years, as the sand is gradually redistributed through natural processes^29^. The total added volume was approximately 21.5 million ${\text{m}}^{3}$, divided over the peninsula (± 17 million $\hbox {m}^3$) and shore-face nourishments (± 4.5 million $\hbox {m}^3$).

**Fig. 6:**
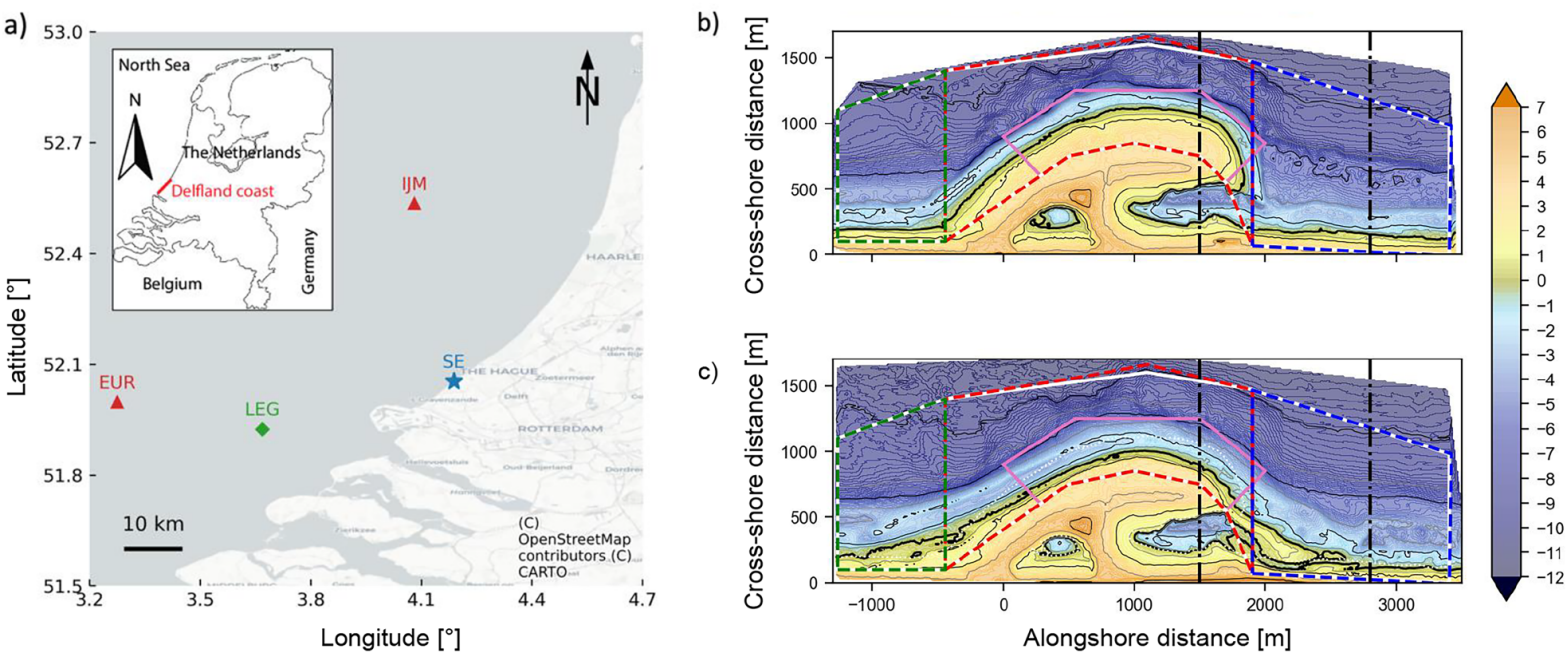
(**a**) Geographical overview of the study region, including the Sand Engine (SE, blue star), the two wave stations (red triangles) at Europlatform (EUR) and IJmuiden (IJM), and the wind station at Lichteiland Goeree (LEG, green diamond). Panel showing the Netherlands is from^48^. (**b**) bed level at August 02, 2011 in m + NAP (NAP is the Dutch reference level, roughly equal to mean sea level), and (**c**) bed level at October 10, 2012 in m + NAP. In panel b and c also show the control areas used for determination of the models performance on different indicators. The bed level control areas are depicted with solid lines: white denotes the area for $BSS_{z}$ and pink for $BSS_{z,SE}$. The volume change control areas $A_{i}$ are indicated with the dashed lines: green denotes area S, red denotes area mid and blue denotes area N. The vertical dash-dot lines indicate the spit area for $BSS_{sl,spit}$.

Near-monthly bathymetric and topographic data of the SE are available since its completion in August 2011^35^. The bathymetric surveys cover an area of 4.7 by 1.6 km and are presented in a local shore-orthogonal coordinate system (Fig. 6). Observed morphological changes were strongest in the first six months (including the storm season of December and January 2011/12), drastically reducing the planform curvature of the shoreline and cross-shore beach slope^35^. After this initial response phase, the morphological changes became slower and more nuanced. Cumulative volume changes over the first 17 months after construction for three areas (South section, peninsula, and North section) showed that 72$\%$ of the sand volume loss around the peninsula had accreted in the adjacent sections (± 1.65 million $\hbox {m}^3$), confirming the natural redistribution of the nourishment^35^. The initially asymmetric shape of the peninsula was reworked during the first months to a near symmetric shape along the coast (Fig. 6b). A sand spit developed at the peninsula’s northern edge, squeezing the lagoon entrance but maintaining an active tidal channel. The morphological changes at the SE in the first period after implementation were strongly correlated to the incoming wave climate^21,35^.

### Delft3D model

We used the Delft3D model suite with the Sand Engine schematization presented by Luijendijk et al.^21^, and later adjusted for model efficiency^5^. It computes sediment transport and corresponding bed level changes resulting from tidal, surge, and wave forcing. The model was used in 2DH mode, meaning a depth-averaged approach in two horizontal dimensions. The model computed values on a curvilinear grid for the hydrodynamic and morphological computations. The grid resolution varied from 35 to 500 m and was highest in the area near the waterline of the Sand Engine. The model was initiated with a bathymetry for the Sand Engine based on the first measurements taken after construction on August $2^{nd}$, 2011. The bathymetry in the remainder of the domain (beyond the 10-m depth contour) was based on surveys of the Dutch Ministry of Public Works, Rijkswaterstaat^49^.

Offshore wave and wind data were used to force the model’s hydrodynamic module. The offshore wave conditions were propagated through the domain using two additional nested grids to increase resolution stepwise to the area of interest. Boundary conditions of the largest grid were derived from measured wave height, period, and direction at two offshore locations (Europlatform and IJmuiden) about 50 km from the SE, combined with a uniform wind field based on the measured conditions at Lichteiland Goeree (Figure 6a). Tidal boundary conditions originated from nesting in a large-scale model for the Dutch Continental Shelf. Finally, time series of observed water levels at Hoek van Holland were used to include local wind surge in the model^21^.

Luijendijk et al.^5^ made several adjustments to increase the computational efficiency of the model schematisation for the Sand Engine presented by Luijendijk et al.^21^. Firstly, they filtered the time series to remove time periods with waves lower than 1 m at the offshore station Europlatform or with wave directions away from the coast, as these did not lead to significant morphological changes. Secondly, the computed morphological changes were accelerated (i.e., scaled) by a factor of three compared to the hydraulic time step^40^. The filtered wave series were compressed by the same factor to match the morphological acceleration.

### Free model parameters

A Delft3D coastal area simulation of morphodynamics contains over 50 model parameters, which, in theory, could all be used to tune the model outcomes and, therefore, contribute to the model’s uncertainty. Varying all these parameters is currently not feasible: one 14-month simulation already takes five days on a high-performance Haswell node (2.6 GHz Intel Xeon E5-2690 v3, 64GB, 1.078 Pflop/s) of the Dutch national supercomputer: SurfSara. Varying 50 parameters with a resolution of 4 values per parameter (which is still relatively coarse) would cost roughly $6 \cdot 10^{30}$ days of computation time ($5 \cdot 4^{50}$)—a (currently) impossible task. The computational budget for this study allowed a total of $\pm 1500$ model runs. As a balanced choice, we focused the uncertainty estimation (GLUE) on the five most influential parameters, with a resolution of four values per parameter, resulting in $4^{5} = 1024$ simulations. To select these five parameters, we started from a list of 30 parameters often cited as important in literature (e.g.^6,21,25,28,50–52^). Next, via expert solicitation, we further reduced the list to 16 parameters (Table 1), categorized into four classes impacting hydraulics, waves, sediment transport, and morphology, based on their implementation in the model^37^. These 16 parameters were then examined through a systematic sensitivity analysis using the Elementary Effects Method (see below) with $\pm 500$ simulations, to reduce the parameter space to the 5 most influential parameters.

The hydraulic parameters influence hydrodynamic processes, such as flow velocities and shear stresses. Here, three parameters were considered. The Chézy coefficient, *C*, which represents the bottom roughness. In the model we used, it is assumed to be uniform over the computational domain. The default value is 65 $\sqrt{m}/s$ and we applied a range between 50 and 80 $\sqrt{m}/s$, based on expert judgment. Next, the horizontal viscosity ($\nu _{h}$) and diffusivity ($D_{h}$) are required for the turbulence model. These are sums of a constant part and a user-defined background value, $\nu ^{back}_{h}$ and $D^{back}_{h}$, respectively. These background values were included in this study. Their default values are zero, but non-zero values have been shown to give better results^21^. Therefore, the applied range was set to 0.1–1 ${\text{m}}^{2} /{\text{s}}$ for both.

The wave parameters influence the propagation and characteristics of the incoming waves. The hydrodynamics of Delft3D are split into two parts, a FLOW module, which includes the roller model, and a WAVE module (SWAN). Here, we focus on four parameters of the roller model. The breaker index, $\gamma$, sets the critical wave height to water depth ratio $H_{s}/h$, from which depth-induced wave breaking starts. The hydrodynamic time step is smaller than the wave update time step, hence the water depth may reduce in between wave time steps. To ensure that the wave height to depth ratio is not exceeded, a maximum allowed value, $\gamma _{max}$, is applied, which enforces wave breaking on the hydrodynamic time-step level. The default value of $\gamma$ is 0.55^37^, but higher values have also been used. The range was set to 0.55–0.8 based on expert judgment. The same range was applied for $\gamma _{max}$ due to its physical similarity to $\gamma$.

We considered two additional parameters that affect wave breaking: the wave energy dissipation coefficient of the roller model, $\alpha _{rol}$, and the mean slope under the roller, $\beta _{rol}$. $\alpha _{rol}$ is a calibration coefficient of *O*(1) that directly scales the energy dissipated by a breaking wave^53,54^. Most studies adhere to the default value of 1, while^25^ examined a range from 0 to 2. $\beta _{rol}$ largely determines the energy transfer to and from the roller (default 0.1). Zero values for $\alpha _{rol}$ and $\beta _{rol}$ result in no energy dissipation and initial test simulations confirmed that this often led to an unstable model. Therefore, the ranges were set to 0.1–2 for $\alpha _{rol}$ and 0.01–0.2 for $\beta _{rol}$.

The sediment parameters directly influence the sediment transport computed by the model. This was the largest group with six evaluated parameters. Four multiplication factors were included that scale the suspended and bed load sediment transport due to currents ($f_{sus}$ and $f_{bed}$) and waves ($f_{sus,w}$ and $f_{bed,w}$). Sediment transport due to the wave-induced alongshore current falls under the current part. The computed sediment transports are multiplied with these factors; hence, their default values are equal to 1. Most studies use similar values for bed and suspended load factors (i.e., $f_{sus}=f_{bed}$, $f_{sus,w}=f_{bed,w}$) (e.g.^21,51^) but occasionally these have been varied independently (e.g.^50^). Predicted sediment transport is often over-estimated and calibrated values hardly exceed 1 (e.g.^21,50,51^). Therefore, the range for the four scaling factors was set at 0.1–1 (a value of 0 would cancel sediment transport altogether).

The grain size of the bed material is represented by the median grain diameter, $d_{50}$. The native grain size $d_{50}$ on the Delfland coast has been estimated at $\pm 250$ $\mu m$^55^, but an analysis during construction of the SE showed an average $d_{50}$ of $281 \mu m$^35^. Huisman et al. 2016^56^ reported significant spatial variation in $d_{50}$ around the SE, specifically coarsening of the sediment in front of the peninsula with + 90 to + 150 $\mu m$ and fining of the sediment in adjacent sections by up to 50 $\mu m$. In the model, $d_{50}$ is assumed uniform over the model domain because the effect of spatial variation is considered of secondary order^57^. However, considering this coarsening and fining with time, the applied range was set to 200–350 $\mu m$. The representative grain size of suspended sediment is determined by multiplying $d_{50}$ with a scaling factor, $fac_{dss}$. The default $fac_{dss}$ is 1 (i.e., the suspended grain size is equal to that of the seabed). The grain size of suspended sediment has been estimated in the range of 60–100% of the grain size of the bed material^58^. We deemed it reasonable to assume that the suspended grain size is not larger than the bed grain size, as this would imply that the largest grains are mobilized by the current while smaller grains stay near the bed. Therefore, the applied range was set to 0.6–1.

Finally, morphology parameters specifically address how sediment fluxes are coupled to the bed elevation changes. Three parameters were considered. The first two scale the effect of stream-wise ($\alpha _{bs}$, default 1) and transverse ($\alpha _{bn}$, default 1.5) bed level gradients on the bed load transport. To model bar dynamics,^25^ have reported realistic values of $\alpha _{bs}$ between 1 and 5. In river engineering, which often includes steep banks, much higher levels have been used (especially for $\alpha _{bn}$, (e.g.^59^)). For coastal purposes, however, the parameters are generally considered at much lower values. The range was set to 1–25 for both parameters to include the possibility of larger values in the SE model. The third morphology parameter is the dry cell erosion factor $\theta _{sd}$, which influences the land-water interface. It enables the erosion of dry cells (defined by a certain water depth threshold) by distributing a fraction ($\theta _{sd}$) of the computed erosion for a wet cell over adjacent dry cells higher up the profile. Luijendijk et al. (2017)^21^ reported this as an important parameter and found the best results for $\theta _{sd} = 1$. The logical range for this parameter, which was applied here, is 0–1.

**Table 1:** Selected free parameters evaluated in the EE analysis with their default value and range. Bold faced variables came out as most influential according to the EE analysis (highest EE ranks) and used in the following step with the GLUE approach. The EE ranks are based on volume changes, shoreline position and bed level.

Class	Parameter	Symbol	Default value	Value of^21^	Range EE (GLUE)	EE Rank	Unit
Hydraulics	Chezy bed roughness	*C*	65	65	50–80	7, > 10, 9	${\text{m}}^{{1/2}} /{\text{s}}$
	Horizontal eddy diffusivity	$D_{h}^{back}$	0	1	0.1–1	> 10, 9, 10	${\text{m}}^{2} /{\text{s}}$
	Horizontal eddy viscosity	$\nu _{h}^{back}$	0	1	0.1–1	> 10, > 10, >10	${\text{m}}^{2} /{\text{s}}$
Waves	**Wave breaking index**	$\gamma$	0.55	0.73	0.55–0.8 (0.55–0.8)	4, 3, 6	-
	Wave breaking limit on time-step level	$\gamma _{max}$	-	0.8	0.55–0.8	10, > 10, > 10	-
	**Roller dissipation coefficient**	$\alpha _{rol}$	1		0.1–2 (1–3)	5, 3, 6	-
	Roller slope	$\beta _{rol}$	0.1		0.01–0.2	6, 8, 8	-
Sediment	**Grain size**	$d_{50}$	-	300	250–350 (200–350)	2, 6, 2	$\mu m$
	Suspended sediment grainsize scaling factor	$f\!ac_{dss}$	1		0.6–1	3, 6, 3	–
	Current related bed and **suspended transport scaling factors**	$f_{sus}$, $f_{bed}$	1	0.5	0.1–1 (0.2–1)	1, 1, 1	–
	Wave related bed and suspended transport scaling factors	$f_{sus,w}$, $f_{bed,w}$	1	0.2	0.1–1	9, 2, 5	–
Morphology	Transverse bedslope parameter	$\alpha _{bn}$	1.5	15	1–25	> 10, > 10, >10	–
	Longitudinal bedslope parameter	$\alpha _{bs}$	1	10	1–25	> 10, > 10, > 10	–
	**Dry cell erosion factor**	$\theta _{sd}$	0	1	0–1 (0–1)	7, 3, 4	–

### Sensitivity analysis (elementary effects)

To identify the five most influential parameters for 1024 simulations and the GLUE analysis, we first examined the importance of a wider set of 16 parameters (Table 1) in predicting bed level, volume, and beach width change for the 14-month study period. We used the Elementary Effects method (EE-method)^60,61^, an effective screening method for models with many parameters, as it requires a relatively low number of computations. The EE-method assesses the sensitivity by varying one parameter at a time at different locations in the parameter space. The method provides insight into the influence, as well as the dependence and non-linearity of the input parameters. The procedure and results are described in more detail in^47,62^. The influence of these parameters is ranked for volume change, shoreline position and bed level in the EE-Rank column of Table 1. The bold-faced parameters indicated in Table 1 were found to be the five highly influential according to these ranks and were selected for the GLUE method, while avoiding including multiple parameters that are closely correlated (e.g. $fac_{dss}$ and $d_{50}$).

### Uncertainty estimation (GLUE)

The Generalized likelihood uncertainty estimation (GLUE) is a tool for estimating uncertainty in model predictions by deriving likelihood distributions of free model parameters, based on a model-observation comparison^46^. An important assumption of the GLUE method is the concept of equifinality^63^, which denotes the possibility that different sets of parameter values can produce predictions with similar skill^64^. A unique optimal parameter set is non-existent, due to a combination of parameter interdependence, model insensitivity^18^, and limitations of the model structure. A drawback of equifinality is that it might be bound to the spatial or temporal calibration domain, as equifinal parameter sets may respond differently outside this domain, notably when forecasting into the future^65^. A way to find a reliable parameter set is to look for the best combined likelihood over several different time periods or locations^10^.

The GLUE method can be criticized for its subjectivity and non-formality (e.g.^10,66,67^). Subjectivity originates from several decisions (behavioral threshold, likelihood definition, included parameters, priors) in the setup of the GLUE analysis. Yet, the advantage of an informal likelihood measure is that too strict rejection of simulations is prevented. As such the GLUE results cannot be used as an absolute measure of uncertainty but do suffice to identify temporal or spatial uncertainty hotspots or assess the relative importance of parameter contributions to the model’s uncertainty. We chose GLUE as it provides a global (in terms of parameter space) extension of the (also subjective) manual calibration process often used in coastal modeling, in which optimal calibration parameters are sought based on manual local improvements of model skill. Therefore, the results are easily extended to lessons for practical model and engineering applications.

**Fig. 7:**
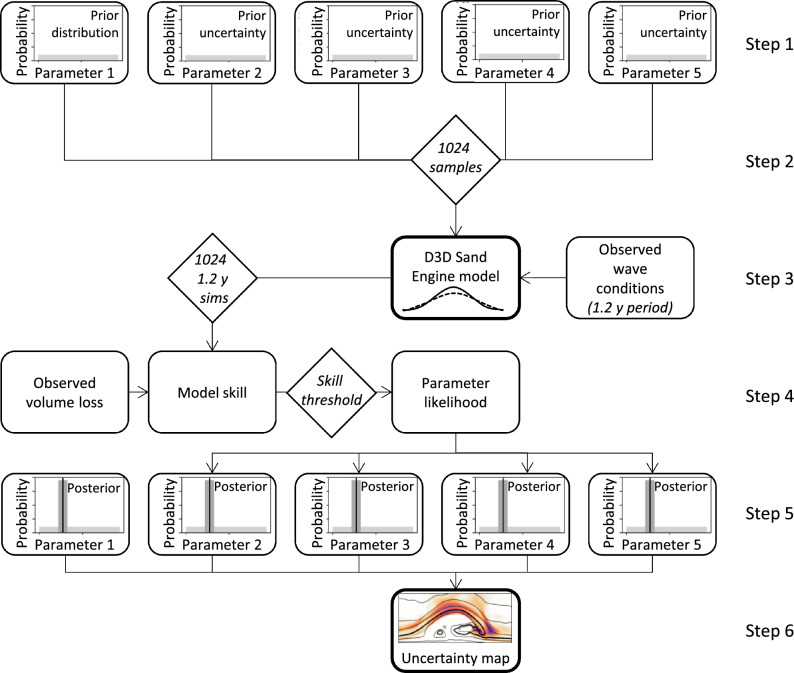
Flow scheme of the GLUE method as applied in this study. Darker colors in the uncertainty map in step 6 indicate areas with more uncertainty.

The GLUE method enables the examination of the model parameter space for acceptable (originally termed behavioral^46^) parameter sets by assigning a non-zero likelihood to all sets with a prediction skill above a predefined behavioral threshold^46^. The first step is to assign prior distributions to each included model parameter (step 1, Fig. 7). Subsequently, a large sample of parameter sets is drawn from the prior distributions (e.g. through Monte Carlo sampling, step 2). For each parameter set, the resulting model prediction (step 3) is evaluated against observation data using a skill score (step 4). The predefined threshold determines whether a model prediction is deemed accepted as a valid solution. Accepted parameter sets are assigned a likelihood value which is scaled based on the predicton skill (non-acceptable sets receive a zero likelihood). The result is a likelihood range for each model parameter, the posterior distribution (step 5), and an observation-based estimation of the parameter uncertainty, determined by the variability in the simulations (step 6).

In the first step, we assumed a uniform prior distribution for each parameter to minimize subjectivity of the procedure. In the second step, 1024 unique parameter sets were drawn from the prior distributions. To achieve equal resolution for each parameter and a well-spread sample, we used a quasi-random sampling method: the Sobol’ low-discrepancy sequence^68^. Discrepancy is a measure of the deviation of the sampled points from a uniform distribution^69^, hence the lower the discrepancy, the more uniformly spread the samples are. The Sobol’ sequence is considered to be superior among the few documented low-discrepancy sampling methods (e.g.^70,71^). As the samples are generated evenly over the parameter space, they have a convergence rate of up to $O(N^{-1})$, whereas for Monte Carlo this is $O(N^{-1/2})$.

In step three, the Delft3D coastal area model was run for the 1024 unique sets of parameter values. In step four, each prediction was compared to observations using a skill metric. In morphological modeling, the Brier skill score (BSS) is a common metric to evaluate model performance^72,73^. The BSS is commonly used to measure the root-mean-squared error (RMSE) of the predicted bed level at each grid point compared to a baseline prediction of zero change:

1 $$\begin{aligned} BSS_{z} = 1 - \dfrac{\sum \limits _{x,y} ({\bar{z}}_{obs}(x,y) - {\bar{z}}_{mod}(x,y))^{2} }{\sum \limits _{x,y} ({\bar{z}}_{obs}(x,y) - {\bar{z}}_{0}(x,y))^{2}} \end{aligned}$$

in which $z_{obs}$ is the observed bed level, $z_{mod}$ the computed bed level, and $z_{0}$ the zero-change baseline.

Considering known limitations of process-based morphological models, the original BSS metric can be a very strict criterion that penalizes the model twice, e.g. for not reproducing dynamic bar behavior or seasonal effects^74^. To relieve the stringency of the BSS, we tailored it to indicate the performance for five different aggregated coastal state indicators: observed cumulative volume changes, cross-shore shoreline position, and point-wise bed level changes. This can be done for the total spatial domain or subsets with specific geomorphology (e.g.^26,75^). All our adapted skill metrics had the same concept as the original BSS: they were RMSE based and used zero change as reference, but they differed in unit (e.g. volume, shoreline position) and/or were constrained by location (erosive area, around water line, etc.).

The first adapted version of the BSS included the bed level changes at the head of the Sand Engine only and is hence referred to as $BSS_{z,SE}$. The model’s ability to predict the shoreline position was evaluated using an adaptation of the BSS where deviations between model and observations due to high temporal and spatial variations in the shoreline positions were neutralized by looking at the averaged position over time:

2 $$\begin{aligned} BSS_{sl} = 1 - \dfrac{\sum \limits _{x,t} (\overline{sl}_{obs}(x,t) - \overline{sl}_{mod}(x,t))^{2} }{\sum \limits _{x,t} (\overline{sl}_{obs}(x,t) - \overline{sl}_{0}(x,t))^{2}} \end{aligned}$$

and a spit focused adaptation $BSS_{sl,spit}$, with $\{x | 1500<x<2800 \}$.

Finally, a volume-based BSS metric was used to evaluate the model’s performance on the prediction of volume changes in different control areas over time:

3 $$\begin{aligned} BSS_{\Delta V} = 1 - \dfrac{\sum \limits _{A,t} (\Delta {\bar{V}}_{obs}(A,t) - \Delta {\bar{V}}_{mod}(A,t))^{2} }{\sum \limits _{A,t} (\Delta {\bar{V}}_{obs}(A,t) - \Delta {\bar{V}}_{0}(A,t))^{2}} \end{aligned}$$

where $\Delta {\bar{V}}(A_{i},t) = \sum \limits _{x,y \in A_{i}} {\bar{z}}(x,y)\cdot {\bar{a}}(x,y)$ and $A_{i}$ represents four control areas used to calculate volume changes defined in Fig. 6b: the middle section of the SE (encompassing the original peninsula), the adjacent sides to the North and South, and the combined SE area, composed of the three sections together. These polygons are similar to those of^35^ except for the middle section, which was adjusted to exclude the subaerial part of the peninsula, where changes are driven by aeolian rather than marine processes.

Next, the threshold separated good or acceptable simulations from those deemed unacceptable. The threshold for each skill definition was chosen such that approximately half of the simulations (500–600) were considered acceptable (Table 2) to have sufficient resolution in the parameter space of the accepted simulations.

**Table 2:** Overview of the applied threshold for each skill definition and the resulting amount of accepted runs (*n*). The bottom three rows show the maximum, mean, and minimum BSS for the respective skill definition.

	$BSS_{\Delta V}$	$BSS_{sl}$	$BSS_{sl,spit}$	$BSS_{z}$	$BSS_{z,SE}$
Threshold	0.75	0.55	0.52	0.43	0.60
n	587	586	589	574	588
Max	0.95	0.79	0.74	0.64	0.88
Mean	0.74	0.54	0.52	0.44	0.59
Min	0.08	0.10	0.14	0.10	0.02

Non-acceptable simulations are assigned a zero likelihood. For each accepted simulation, the likelihood was computed from the skill value:

4 $$\begin{aligned} L_{BSS,i} = \dfrac{BSS_{i}}{\sum _{i=1}^{n} BSS_{i}}. \end{aligned}$$

In which $i=1,\ldots ,n$ are the ranked and accepted simulations. With the different likelihood measures, a combined likelihood *CL* was established and computed as the product of the likelihoods for the different evaluated results.

5 $$\begin{aligned} CL_{j} = \left( \prod _{i=1}^{N_{L}} L_{i,j} \right) ^{\dfrac{1}{N_{L}}} \end{aligned}$$

In which the combined likelihood of simulation *j* is computed as the product of N_L_ likelihood values. Because this is a multiplicative method, if any of the likelihood values is zero, the combined likelihood is also zero. Using the combined likelihood forces the model to behave fair on all aspects (bed level, shoreline, volume). Still, if a certain aspect is considered more important than another, it can also be a choice to focus only on the skill of that aspect. This can lead to a different set of acceptable parameter settings.

In step five, the (combined) likelihood scores were used to transform the prior (uniform) parameter distributions to marginal posterior distributions (effectively a likelihood distribution for each parameter). The posterior distributions were then compared with the prior distributions and the default or reference values for the parameters. The influence of each input parameter on the prediction was quantified using the Kolmogorov–Smirnov (K–S) distance^36^, which denotes the largest distance between the prior and the posterior distributions. The larger the K–S distance, the more influential the parameter. In addition, the posterior distributions were used to estimate several optimal parameter sets (OPS). The model was then run for each OPS to evaluate whether they outperformed the other simulations.

In the final step, we defined uncertainty bounds through the width of the 90% confidence interval ($W_{CI90\%}$) derived from all accepted simulations. As the results of the different simulations were non-Gaussian distributed, we defined the 90% confidence interval between the 5th and 95th percentile of the empirical cumulative density function. The width $W_{CI90\%}$ was derived by subtracting the 5th from the 95th percentile.

### Importance of model parameter uncertainty

To put the importance of model parameter uncertainty in perspective, we compared the results to the variation in outcomes introduced by year-to-year variations in wave forcing. To this end we performed eight exploratory simulations with different wave forcing time series. We selected eight years with different wave forcing magnitudes and chronologies from 25 years of historic wave data. The selection was made based on the total wave energy density at − 10 m + MSL depth contour (low, medium, high with respect to the 25 year average) and dominant direction (west or north). The model parameter values were kept equal to the OPS parameter values in all these simulations.

## Data Availability

The morphological data of the Sand Engine presented in this study are available at the 4TU.Centre for Research Data: 10.4121/collection:zandmotor.
